# Clinical Implications of COVID-19 Presence in CSF: Systematic Review of Case Reports

**DOI:** 10.3390/cells11203212

**Published:** 2022-10-13

**Authors:** Ibrahim Elmakaty, Khaled Ferih, Omar Karen, Amr Ouda, Ahmed Elsabagh, Ahmed Amarah, Mohammed Imad Malki

**Affiliations:** College of Medicine, QU Health, Qatar University, Doha P.O. Box 2713, Qatar

**Keywords:** SARS-CoV-2, systematic review, cerebrospinal fluid, CNS, infections

## Abstract

This systematic review focused on severe acute respiratory syndrome coronavirus 2 (SARS-CoV-2) patients that had detected SARS-CoV-2 virus in cerebrospinal fluid (CSF). A systematic literature search was carried out in PubMed, Embase, Scopus, Web of Science, Medrxiv, and Biorxiv databases from inception to 19 December 2021. Case reports or case series involving patients with proved SARS-CoV-2 presence in CSF by polymerize chain reaction were included. Our search strategy produced 23 articles documenting a total of 23 patients with positive SARS-CoV-2 in the CSF. Fever (55%) was the most common symptom, followed by headaches (41%), cough (32%), and vomiting/nausea (32%). The majority of the cases included was encephalitis (57%), 8 of which were confirmed by magnetic resonance imaging. The second most prevalent presentation was meningitis. The cerebral spinal fluid analysis found disparities in protein levels and normal glucose levels in most cases. This study demonstrates that SARS-CoV-2 can enter the nervous system via various routes and cause CNS infection symptoms. SARS-CoV-2 has been shown to infect the CNS even when no respiratory symptoms are present and nasopharyngeal swabs are negative. As a result, SARS-CoV-2 should be considered as a possible cause of CNS infection and tested for in the CSF.

## 1. Introduction

In December 2019, the first cases of coronavirus disease 2019 (COVID-19), a disease related to severe acute respiratory syndrome coronavirus 2 (SARS-CoV-2), were identified in China [1]. The virus has continued to spread since then, and the World Health Organization (WHO) declared COVID-19 a pandemic on 11 March 2020 [2]. Since the end of 2019, the new coronavirus SARS-CoV-2 has infected hundreds of thousands of people all over the world. More than 281.8 million cases were reported worldwide as of 29 December 2021, with more than 5.4 million fatalities [3]. Patients with severe COVID-19 can quickly develop acute respiratory distress syndrome, and the majority of these deaths was related to severe respiratory failure [4]. Studies all around the world reported a wide spectrum of signs and symptoms associated with SARS-CoV-2, including dyspnea, non-productive cough, fever, myalgia, fatigue, diarrhea, and nausea/vomiting, while some patients are known to be asymptomatic [5].

A preliminary retrospective study of 214 hospitalized patients found that 36% of them had neurological symptoms [6]. Multiple neurological signs, such as headaches or defined neurological illnesses, such as Guillain–Barré syndrome or encephalitis, have been linked to SARS-CoV-2 infection in subsequent articles and series [7]. According to research, more than 35% of COVID-19 individuals develop neurological symptoms, and some COVID-19 patients may present with neurological symptoms as their first symptom [8]. SARS-CoV-2 has been proven to elicit several alterations to the cerebral spinal fluid (CSF) content, including an increase in white blood cell counts and protein [9]. There is a lot of evidence in the literature of COVID-19 crossing the blood–brain barrier (BBB) and entering the central nervous system (CNS), producing CNS-related symptoms either directly or indirectly through circulation [10]; however, there are only a few examples where SARS-CoV-2 has been found in CSF samples [11,12]. Coronaviruses have been known to infiltrate the CSF in the past, as evidenced by the instance of a positive human Coronavirus OC43 in the CSF discovered by polymerase chain reaction (PCR) in 2004 [13].

The main objective of this systematic review of case reports is to define the change in CSF composition, presentations and outcomes in clinical scenarios of patients with confirmed PCR SARS-CoV-2 viral ribonucleic acid (RNA) presence in CSF.

## 2. Materials and Methods

### 2.1. Protocol and Registration

The Preferred Reporting Items for Systematic Reviews and Meta-Analyses (PRISMA) guideline was used to record this systematic review (see Table S1 in Supplementary Materials) [14]. The protocol of this systematic review was registered with the international prospective register of systematic reviews (PROSPERO) online database (PROSPERO Identifier: CRD42022301576).

### 2.2. Search Strategy

We developed our search strategy in PubMed using Medical Subject Headings (MeSH) terms for COVID-19 and central nervous system infections in case reports and case series study types. Then, Polyglot translator was used to transfer the developed search strategy to Embase, Scopus, and Web of Science [15]. Similar terms were also used to search Medrxiv and Biorxiv. The full search strategy for each database is available in the Supplementary Materials. All search results were then exported to EndNote X7, where duplicates found by the software were removed. The remaining articles were uploaded to the Rayyan platform for screening [16].

### 2.3. Eligibility Criteria

We included case reports, case series, or letters that met the following criteria: (1) cases with CNS inflammation (2) with confirmed presence of SARS-CoV-2 RNA in the CSF using PCR (not SARS-CoV-2 antibodies in CSF) (3) without a confirmed co-infection with another pathogen in the CSF (4) and in a clinical scenario (not an autopsy/animal). We accepted any diagnostic criteria presented in the included article to characterize CNS disorders because various studies had varying criteria for defining CNS disorders. Encephalitis should be detected using magnetic resonance imaging (MRI), an electroencephalogram (EEG), or CSF analysis while a computed tomography (CT) scan is only accepted if there is clear evidence and MRI was not performed, whereas encephalopathy can only be diagnosed using physical signs, according to the included article. Meningitis diagnosis must be confirmed either by CSF analysis or MRI. Included studies were not restricted to patient demographics, and there were no language or publication date restrictions. We excluded cases where CNS inflammation was due to any SARS-CoV-2 vaccines. Google Translator was used to translate all articles written in a foreign language into English when needed.

### 2.4. Study Selection and Screening

The records obtained from the literature search were further evaluated using the Rayyan platform to screen titles and abstracts [16]. Titles and abstracts were screened independently by two reviewers, and any disagreements were resolved by consensus among the entire team. The full texts of studies that were deemed potentially eligible were then retrieved and double-screened independently, with any discrepancies referred to a co-reviewer. 

### 2.5. Data Extraction

We extracted data from each eligible study on patient demographics (age, sex, ethnicity), country, presenting symptoms, physical examination, method of SARS-CoV-2 diagnosis, CSF analysis (color, protein, glucose, cells morphology, culture), other lab investigations, diagnosis, and outcome. Data extracted from each study was conducted by two reviewers independently, with any inconsistencies referred to a third reviewer. All of the data were summarized and compiled into an online Excel spreadsheet that was accessible to all of the authors.

### 2.6. Quality of Studies

Case reports are by their nature biased. In systematic reviews of case reports, however, standardized tools have been created to assess their methodological quality. To assess the quality of the case series and reports included in the study, we used Murad et al.’s modified Newcastle–Ottawa Scale (NOS) [17]. Selection, ascertainment, causality, and reporting are the four domains assessed by this tool in a series of 8 questions. Questions 4,5 and 6 in the tool were left out because they are largely applicable to cases of adverse medication reactions as described by the tool [17] and do not relate to our topic. Based on their cumulative score in the remaining 5 questions, the articles’ risks of bias were classified as “high risk”, “medium risk”, or “low risk”. If case reports or case series scored 4 or 5 points on the quality assessment questions, we considered them to have a low risk of bias. We considered articles with a score of 3 to have a medium risk of bias, and those with a score of less than 3 to have a high risk of bias. 

### 2.7. Data Analysis

Data extracted from each article were summarized and presented in a table. The cases were then described narratively in the text to combine and highlight the similarities between them and, where possible, to draw conclusions. Due to the descriptive nature of this systematic review and the small number of cases, we employed descriptive statistics to present demographics and clinical characteristics. Means were used to report continuous variables, while using frequencies and percentages were used for dichotomous variables. 

## 3. Results

### 3.1. Study Selection

**Figure 1:**
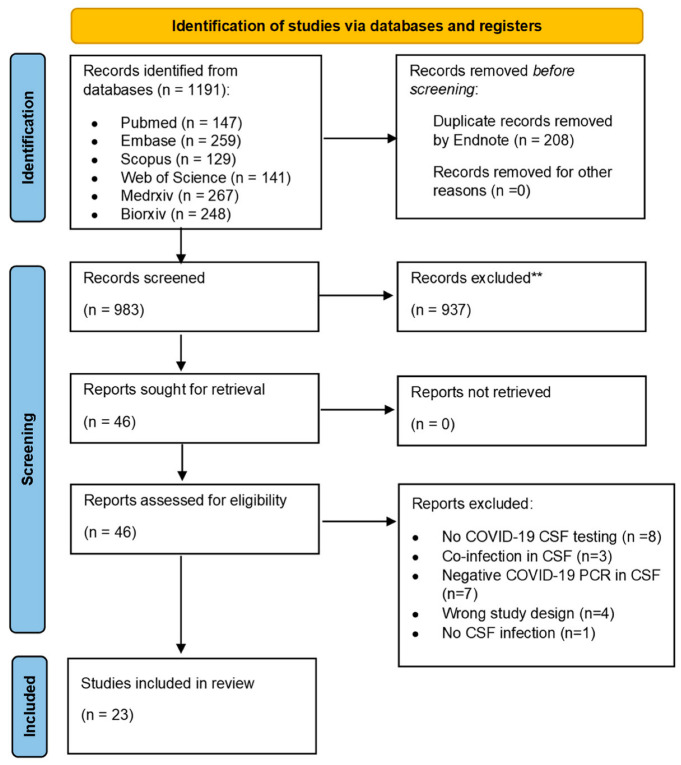
PRISMA flow-chart of the study selection process.

Figure 1 is a PRISMA flow diagram that shows the process of study selection. Our search strategy yielded 1191 references. Of those, 208 duplicates were removed by EndNote, and the remaining 983 records were screened for title and abstract. The title and abstract were available in English for all those articles, while the full text for nine articles was in foreign languages (five Spanish, two Hungarian, two Russian). A total of 937 articles was excluded after the title and abstract screening, leaving 46 studies for full text screening. All 46 records’ full-texts were obtained and screened for eligibility. Twenty-three articles were excluded for the reasons shown in Figure 1 (citation of those records and full reasoning for exclusion is provided in Table S2 in Supplementary Materials), and the remaining 23 articles were included in our data synthesis [11,12,18,19,20,21,22,23,24,25,26,27,28,29,30,31,32,33,34,35,36,37,38].

### 3.2. Study Characteristics and Patient Demographics

The 23 articles that met our inclusion criteria reported on 23 individual patients from 12 different countries. Table 1 summarizes the characteristics and extracted data from the included studies. Most articles were case reports 20 (87%), there was one patient in a case series (4.3%), and one case was in a letter to editor (4.3%). All studies were available in English, and most cases were reported from Iran with seven cases (30.4%), Brazil with four cases (17.4%), and United Arab Emirates (UAE) and the United States of America (USA) with two cases (8.7%) each. Out of those 23 individuals with positive SARS-CoV-2 CSF, the age ranged from 9 to 70 with a mean of 40.7 years, with the majority (15) being male (65.2%). 

### 3.3. Clinical Characteristics of Patients with Confirmed COVID-19 in CSF

Information about COVID-19 presenting signs and symptoms in children was available for all the cases. Of the 23 cases, 14 had fever (61%), 7 cases had headaches (39%), 7 patients had cough (30%), 5 cases had vomiting/nausea (22%), 3 had fatigue (13%), 2 cases had myalgia (9%), 2 had shortness of breath (9%), and no one had upper respiratory symptoms, rhinorrhea, sneezing, or nasal congestion. No studies reported data points on the presence or absence of pediatric anosmia or dysgeusia. 

Thirteen of the 23 (57%) patients were identified with SARS-CoV-2-associated encephalitis. Three instances were diagnosed as meningoencephalitis, one case as acute necrotizing encephalopathy, one case as anti-N-methyl-D-aspartate (NMDAR) encephalitis, one case as limbic encephalitis, one case as encephalomyelitis, and the other six cases as encephalitis without a defined classification. Eight of the 13 instances were identified using brain MRI, one using brain CT, two using CSF analysis, one using EEG, and one using both CSF analysis and EEG. There were two cases of encephalopathy without evidence of encephalitis. One of them showed normal neuroimaging, while in the other case, MRI was not done. There were four single occurrences of meningitis, one of which had meningeosis carcinomatosis. Two patients were diagnosed using CSF analysis, one through MRI, and one through combined MRI and CSF analysis. The remaining four cases were acute transverse myelitis confirmed by Spinal MRI, acute cerebellitis diagnosed by brain MRI, brainstem encephalitis diagnosed by brain MRI, and demyelinating syndrome with normal neuroimaging. More details on the diagnoses and neuroimaging findings can be found in Table 1. Of the studies with hospital discharge data, 13 out of 23 patients were discharged (72.6%). The average length of stay ranged from 7 to 60 days (the mean length of stay was 28 days). 

Table 2 displays the time elapsed between the onset of the first clinical symptoms and SARS-CoV-2 PCR testing, as well as the reported PCR results and genes tested by PCR. In four cases, the CSF analysis was indicated and performed after the nasopharyngeal (NP) swab, whereas in seven other cases, both the CSF analysis and the NP swab were performed, and both were positive. Despite being positive in all CSF analysis, the NP swab was negative for SARS-CoV-2 in 8 of the 23 cases. The time elapsed in Table 2 clearly shows that if respiratory symptoms develop, neurological symptoms follow, as some patients did not report any respiratory symptoms. The cycle threshold (CT) value was reported in 1 case out of 15 positive NP PCR swabs tested, and in 6 of 23 SARS-CoV-2 positive CSF samples. The exact CT values for each reported positive case are shown in Table 2.

### 3.4. Changes in CSF Associated with Confirmed COVID-19 in CSF

Table 3 describes CSF analysis results for the included cases. Out of the 23 documented cases, 8 (34.8%) described the collected CSF. All were clear and colorless characteristics of viral CSF presentation except in two case reports where they were described to have a pink color. Protein concentrations in CSF analysis were mentioned in 19 (87.0%) articles; 8 of those had high protein content (more than 60 mg/dL), 3 were below 15 mg/dL, and the remaining 7 cases had normal protein levels. CSF glucose levels were given in 13 (59.1%) cases; the majority (10 cases, 76.9%) was within the normal CSF glucose range between 50 mg/dL to 80 mg/dL, while it was borderline (12 mg/dL, 45 mg/dL and 90 mg/dL) in the remaining 3 cases. White blood cell (WBC) count was stated in 15 (65.2%) cases. Only 3 cases were within the normal range (0–5 cells/µL), while it was elevated in the remaining 12 cases, with lymphocyte dominance whenever the morphology of the cells was described as typical for viral CSF infections. On the other hand, six (26.1%) articles mentioned the presence of red blood cells (RBCs) in the CSF. All CSF cultures and PCR were negative for all tested bacteria and viruses except for COVID-19.

### 3.5. Quality Assessment

Using Murad et al.’s standardized tool for assessing case reports and case series quality [17], 6 (26.1%) of the studies had a high risk of bias, 6 (26.1%) had a medium risk, and 11 (47.8%) had a low risk of bias. There was a total of 10 (43.5%) studies that failed to provide adequate assertion for our outcomes of interest (CSF analysis), while most studies 18 (78.2%) provided clear adequate information about the exposure (method of SARS-CoV-2 diagnosis). Seven (30.4%) did not make enough follow-up time for the clinical outcome of the cases to be fully clear. Full quality appraisal for each article is available in Table S3 in the Supplementary Materials.

## 4. Discussion

### 4.1. Principal Findings

**Figure 2:**
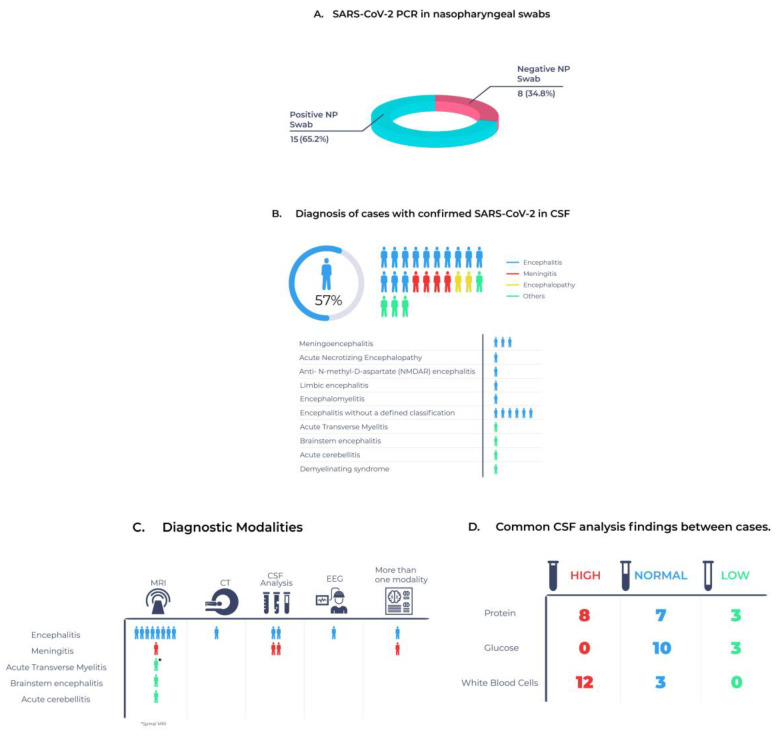
Visual summary of the significant findings in (**A**) results of SARS-CoV2 PCR in nasopharyngeal swabs, (**B**) diagnosis reached for cases with confirmed SARS-CoV-2 in CSF, (**C**) modalities for confirming the diagnosis, and (**D**) common CSF analysis findings between cases.

In this systematic review, we found that most of the patients presented with fever, headache, and cough. Most of the included cases were encephalitis, 13 of which were confirmed by MRI. Meningitis was the second most common presentation. The majority of these patients exhibited colorless CSF with large quantities of leukocytes with dominating lymphocytes, normal glucose levels, and changes that are commonly seen in viral CSF infections. Furthermore, several individuals tested negative for SARS-CoV-2 on a nasopharyngeal swab but positive for SARS-CoV-2 in the cerebral fluid when PCR was performed. Figure 2 summarizes the main findings of our study. Moreover, the data revealed that alternative therapy was used in situations where doctors diagnosed SARS-CoV-2-related nervous system illness. As a result, further research is needed to develop a clear management strategy in the event of a SARS-CoV-2 nervous system infection diagnosis.

Despite the fact that our systematic review concentrated on positive SARS-CoV-2 CSF results, the vast majority of patients with SARS-CoV-2 associated CNS inflammations had negative SARS-CoV-2 CSF PCR. One study, for example, summarized the findings of various case reports and series and found that 8 cases of encephalitis and 19 cases of Guillain–Barré syndrome were reported out of 901 individuals, with just a handful having positive SARS-CoV-2 CSF findings [39]. Another systematic review and meta-analysis focused on patients with encephalitis as a complication of SARS-CoV-2 found that while the incidence of encephalitis in the general population of hospitalized SARS-CoV-2 patients was low at 0.215%, the mortality rate of patients with encephalitis as a complication of SARS-CoV-2 was high at 13.4% [40]. These encephalitis cases were among the critically sick SARS-CoV-2 patients who had abnormal clinical parameters such as elevated blood inflammatory markers and cerebrospinal fluid pleocytosis [40]. It is also crucial to highlight that the incidence of positive SARS-CoV-2 CSF is a small portion of the estimated 0.215% incidence of the encephalitis in the general population of hospitalized SARS-CoV-2 patients, and that the presence of SARS-CoV-2 in the CSF does not guarantee an encephalitis diagnosis if there is no indication of brain inflammation. We found no evidence that SARS-CoV-2 infections with positive CSF findings are limited to those with severe diagnoses. Positive CSF for SARS-CoV-2 did not particularly cause a worse diagnosis, as was evident in our results section, and those 23 cases did not have unique features. This means that even a simple patient case of suspected encephalopathy without changes in neuroimaging can present with positive SARS-CoV-2 virus in CSF analysis.

### 4.2. COVID-19 CSF Entry Mechanisms

Although primary data on SARS-CoV-2-related CNS inflammation are few, making inferences regarding the molecular characteristics and pathophysiology of encephalitis in SARS-CoV-2 problematic at this time, there are numerous postulated pathways underlying the pathophysiology of CNS inflammation as a SARS-CoV-2 consequence.

The first suggested mechanism is the direct invasion of the SARS-CoV-2 virus into the CNS and brain parenchyma by trans-synaptic propagation or hematogenous invasion [40]. Hematogenous invasion occurs when SARS-CoV-2 enters the brain parenchyma by hematogenous invasion after crossing the BBB. SARS-CoV-2 begins by invading vascular endothelial cells that exhibit the angiotensin-converting enzyme 2 (ACE2) receptor. It then binds with ACE2 on nearby neurons, glial cells, and other vascular cells, initiating a viral budding cycle [41]. Through this interaction, SARS-CoV-2 can breach the BBB, allowing the virus to enter the CNS by causing damage to both vascular and neuronal tissue [40]. Another hypothesized method for SARS-CoV-2 hematogenous invasion of the CNS is an infection of white blood cells in the bloodstream, since many lymphocytes, monocytes, and granulocytes are susceptible to SARS-CoV-2 due to their expression of the ACE2 receptor [42]. After becoming infected with SARS-CoV-2 in the bloodstream, immune cells can cross the BBB, entering the CNS and carrying the SARS-CoV-2 virus with them, where they can infect other cell types inside the CNS, resulting in brain inflammation and infection [42]. Alternatively, SARS-CoV-2 can bind to the angiotensin II receptor on the cell membrane of peripheral nerve cells during trans-synaptic propagation and enter cells via receptor-mediated endocytosis [40]. It then travels retrogradely to the CNS using active axonal machinery [42]. For instance, SARS-CoV-2 can invade the olfactory primary sensory neurons and go to the cribriform plate of the ethmoidal bone through the olfactory epithelium. It then enters the anterior cerebral fossa and can spread throughout the brain parenchyma, producing a brain infection [41]. This direct invasion mechanism is what our results mostly align with, though it is the least supported in the literature, as most documented brain infection cases are CSF-negative for SARS-CoV-2 [40]. Alternatively, during trans-synaptic propagation, SARS-CoV-2 can attach to the angiotensin II receptor on the cell membrane of peripheral nerve cells and enter cells by receptor-mediated endocytosis [40]. It then goes back to the CNS through active axonal machinery [42]. SARS-CoV-2, for example, can infiltrate the olfactory primary sensory neurons and go to the cribriform plate of the ethmoidal bone via the olfactory epithelium. It subsequently penetrates the anterior cerebral fossa and has the potential to spread throughout the brain parenchyma, resulting in brain infection [41]. Our findings are mainly consistent with this direct invasion mechanism; however, it is the least supported in the literature because most reported brain infection patients have CSF that is SARS-CoV-2-negative, while we in this systematic review included only SARS-CoV-2 CSF-positive patients [40]. It is worth noting that the absence of virus in CSF does not rule out a direct viral invasion, as shown by other infectious disorders such as West Nile virus or enterovirus infections [43].

The systemic inflammation generated by the SARS-CoV-2 virus is another possible explanation for the pathogenesis of CNS inflammation as a consequence of COVID-19 [44]. Infection with SARS-CoV-2 stimulates the innate immune system, resulting in the creation of huge amounts of inflammatory cytokines, known as the cytokine storm, which causes systemic inflammatory response syndrome [44]. After that, the inflammatory cytokines are carried throughout the bloodstream to many different bodily systems, including the CNS, where they cause nervous system dysfunction and brain inflammation [40]. In a prospective multicenter analysis of 25 cases of encephalitis caused by SARS-CoV-2 infection, the majority of patients had clinical, imaging, and CSF results pointing to a cytokine-mediated mechanism, whereas some cases were more likely to be caused by immuno-mediated pathways [45]. This is substantiated by the presence of proinflammatory cytokines in CSF analysis provided by this systematic review. Molecular mimicry is the final suggested mechanism of CNS inflammation. This occurs as a result of the main SARS-CoV-2 infection in the body, where there is an increase in host antibodies and lymphocytes [41]. Despite the fact that these immune molecules are meant to be specific for SARS-CoV-2 viral antigens, some of them are cross-reactive and can possibly assault self-antigens [40]. Following that, widespread CNS injury occurs as a result of damage to the vascular endothelium and brain parenchyma, resulting in brain inflammation [41]. There are examples of Guillain–Barré syndrome, which is known to develop via molecular mimicry, further supporting the notion of molecular mimicry as the pathophysiology of encephalitis as a consequence of COVID-19 [46]. This is substantiated by a meta-analysis, which showed that autoimmune encephalitis is the most prevalent type of encephalitis in COVID-19 [41].

### 4.3. Other CSF Changes in SARS-CoV-2

Some CSF analyses in the included cases revealed some changes that might be associated with SARS-CoV-2 infection in the CNS. In a study of 25 patients of SARS-CoV-2 encephalitis, CSF examination revealed hyperproteinorrachia and/or pleocytosis in 68% of cases [45].

One study marked an increase in neurofilament light (NfL) [19], which could be a marker for neurodegeneration and correlate with the presence of cognitive impairment [47]. This article also described the presence of tau proteins in the CSF [19]. Another common finding in CNS SARS-CoV-2 infection is the occurrence of immune complexes and other pro-inflammatory cytokines such as interleukin-1, interleukin-6, immunoglobulin G (IGg), and ferritin [19,30,36,38,48]. SARS-CoV-2 is also associated with increased angiotensin-converting enzyme (ACE) in the CSF [48], which could be related to the hypothesized entry pathway for SARS-CoV-2 [49]. Furthermore, SARS-CoV-2 CNS infection shows an increase in the CSF high-sensitivity C-reactive protein (hsCRP) and oligoclonal band levels [50]. However, analysis of the CSF in COVID-19 negative patients has identified the presence of antibody markers against COVID-19 [50].

### 4.4. Issues in Current Practice

Multiple case reports in the literature have reported that lumbar puncture is contraindicated, and therefore CSF could not be obtained for SARS-CoV-2 testing [51,52]. In addition, there were many cases where lumbar puncture was performed but CSF was not tested for SARS-CoV-2 without providing justifications for not testing [53,54,55,56]. Other case studies reported that CSF samples were not tested for COVID-19 because there is no Food and Drug Administration (FDA) approved PCR kit or a commercially available kit for SARS-CoV-2 diagnosis in CSF [57,58]. While some authors requested CSF SARS-CoV-2 PCR for the laboratory in their country/hospital, it was denied [59,60]. On the other hand, many reported testing the CSF with SARS-CoV-2 PCR and found negative results despite COVID-19 being the only possible cause for the underlying pathology [50,61,62]. In such cases, the underlying neuropathogenesis is probably due to an autoimmune cause, which may possibly suggest better treatment and response to steroids [63]. However, SARS-CoV-2 cannot be ruled out completely, as those negative results could be false negatives. False negative results are due to a low amount of viral SARS-CoV-2 particles that are insufficient for detection, delay in testing the CSF for COVID-19 after the onset of symptoms, or because of transient viremia [64,65]. We suspect that the prevalence of SARS-CoV-2 in the CSF is higher than what we present here because many people did not undergo SARS-CoV-2 CSF PCR, either because lumbar puncture was not recommended or because there was no methodology for conducting SARS-CoV-2 PCR in CSF fluid. False positives are highly unlikely, as PCR testing for SARS-CoV-2 has near-perfect specificity [66]. 

### 4.5. Policy Implications and Future Research

To give prompt and effective care to patients with COVID-19 CNS-related infection, guidelines for early imaging and CSF analysis should be developed. Lumbar puncture must be done when the risk of CNS-related COVID-19 is high as indicated by severe signs and symptoms of CNS infections and the presence of CNS hyperintense lesions or leptomeningeal enhancement in imaging [67,68]. Moreover, there is a clear need for SARS-CoV-2 CSF testing to be broadly accessible in hospital settings [69]. Therefore, we suggest making a PCR kit specifically for CSF testing that is more reliable and that has an appropriately adjusted limit of detection. Those SARS-CoV-2 CSF PCR kits can be further added to the currently available CSF viral PCR panels to make the testing procedure more feasible for clinicians. Finally, much research can be done retrospectively on stored CSF samples to give a much clearer picture on the prevalence and clinical presentations when SARS-CoV-2 penetrates the CSF. Thus, we would also want to encourage doctors to gather and keep CSF samples from COVID-19 patients for future investigation and research purposes.

### 4.6. Strength and Weaknesses

To our knowledge, this is the first study that focuses mainly on the presence of SARS-CoV-2 in the CSF and that has attempted to combine all available evidence in the literature in a systematic methodology with a standardized appraisal of quality in included cases. Therefore, our review study is an essential first step toward a better understanding of the CSF changes and consequences accompanied by SARS-CoV-2 CSF infiltration. To conduct more rigorous research, epidemiological studies can expand on the findings presented here. This narrative synthesis of case reports has a number of drawbacks. Firstly, case reports are inherently subjective, provide a non-random sample, and in many cases do not allow for causality claims [70]. In many cases, there was a lack of detailed information on patient outcomes. Moreover, this systematic review relied on the current literature of a small sample of 23 cases that did not allow for a more robust quantitative synthesis. Further, there is a lack of generalizability because demographics and baseline data cannot be utilized to predict results in a wider population. Finally, despite searching preprint databases such as Medrxiv and Biorxiv, publication bias cannot be completely eliminated because more difficult cases are more likely to be recorded and published, resulting in many cases being missed. Finally, we had little control over the reported CSF marker because this was a systematic review rather than a prospective trial; hence, our findings on CSF analysis were restricted.

## 5. Conclusions

This study shows that SARS-CoV-2 can be present in the nervous system via different routes, and it can present with CNS infection symptoms. SARS-CoV-2 has shown the ability to infect the CNS even when there are no respiratory symptoms and nasopharyngeal swabs are negative. Hence, SARS-CoV-2 should be considered as a possible cause of CNS infection, and testing for it in the CSF should be conducted.

## Figures and Tables

**Table 1:** Characteristics of included confirmed COVID-19 CSF cases.

First Author	Article Type	City, Country	Age, Sex Ethnicity	Presenting Symptoms	Physical Exam	COVID-19 Swab	Other Test Result	Diagnosis	Outcome
Yousefi et al. (2021) [18]	Case report	Imam Hassan Hospital, Iran	9-year-old, Iranian Turkish Girl	Fever, headache, low back pain	Head and neck stiffness, +Brudzinski, +Kernig.	Negative PCR in NP swab	CBC: ↑LDH, ↑WBCs, 88% neutrophils.	Meningitis after COVID-19 infection by CSF analysis	Discharged after 10 days of hospitalization with follow-up
Virhammar et al. (2020) [19]	Case report	Sweden	55-year-old, Woman	Day1: fever, myalgia. Day 7: lethargic, unresponsive	Stable, stuporous, multifocal myoclonus. No respiratory problems.	Positive PCR in NP swab	MRI: pathologic signal symmetrically in central thalami, medial temporal lobes, and brain stem.	Acute necrotizing encephalopathy with COVID-19	Extubated on day 35 and discharged to rehabilitation
Steininger et al. (2021) [20]	Case report	Germany	53-year-old, Man	Fever, headache	Meningism, decreased vigilance. No respiratory problems.	Positive PCR in NP swab	CBC: ↓WBCs, ↓neutrophil, normal CRP. MRI: infratentorial and supratentorial lesions.	Meningeosis carcinomatosis with COVID-19 meningitis	Chemotherapy after viral clearance
Shahali et al. (2021) [21]	Case report	Iran	63-year-old, Caucasian Man	Loss of control in lower limbs, absent sensation below chest, constipation, urinary retention	91% O2sat, hypotonic lower limbs, hypoesthesia below T8.	Positive PCR in NP swab	Brain CT and MRI: normal. Spinal MRI: increased T2 signal involving central gray matter and dorsal columns.	COVID-19-associated acute transverse myelitis	Neurologic symptoms cleared after 1 week, discharged after 5 weeks
Fadakar et al. (2020) [22]	Case report	Iran	47-year-old, Man	Pain, progressive vertigo, headache, ataxia, fatigue, pain, cough (10 days)	Ataxic gait, dysarthria, impaired tandem gait head titubation, truncal swaying, dysarthria, saccadic pursuit, loss of optokinetic nystagmus, dysmetria.	Positive PCR in NP swab	CBC: normal, ↑ferritin level. Brain MRI: hyperintensities in cerebellar hemispheres and vermis. FLAIR: edema, cortical–meningeal enhancement.	Acute cerebellitis associated with COVID-19	Improvement of vertigo after 14 days. After 1 month, his ataxia improved
Domingues et al. (2020) [12]	Case report	Brazil	42-year-old, Woman, in São Paulo	Paresthesia in left upper limb, later: left hemithorax, hemiface	Hypoesthesia, coryza, nasal obstruction.	Negative PCR in NP and nasal swabs	CBC: normal. Chest tomography: normal. Brain MRI: normal.	Demyelinating disease, COVID-19-associated	Full recovery after 3 weeks.
Huang et al. (2020) [23]	Letter to the Editor	Downtown Los Angeles	40-year-old, Woman	Fever, syncope	Awake, alert, lethargic but coherent, neck stiffness, photophobia.	Positive PCR in NP swab	Non-contrast head CT: normal. Chest X-ray: clear. EEG: generalized slowing with no epileptic discharges. No MRI.	COVID-19 encephalitis without MRI confirmation	Improved mental status at hospital day 12
Moriguchi et al. (2020) [11]	Case report	Yamanashi University Hospital, Japan	24-year-old, Man	LOC, lying on the floor in vomit (day 9)	Neck stiffness, transient generalized seizures.	Negative PCR in NP swab	Brain MRI: hyperintensity in wall of right lateral ventricle and hyperintense signal changes in the right mesial temporal lobe and hippocampus.	COVID-19 meningitis based on MRI	Discontinued treatment at day 15
Khodamoradi et al. (2020) [24]	Case report	Iran	49-year-old, Woman	Chills, fever, nausea, vomiting, malaise	Awake, alert, oriented, febrile (38 °C).	Negative PCR in NP swab	CBC: normal. Chest CT: normal.	COVID-19 meningitis based on CSF analysis	Discharged at day 21 after improvement
Al-olama et al. (2020) [25]	Case report	Dubai, United Arab Emirates	36-year-old, Male	Fever, headache, body pain, cough, diarrhea, vomiting	Pharyngitis.	Positive PCR in NP swab	Brain CT: frontal intracerebral hematoma with subarachnoid hemorrhage. No MRI.	Meningoencephalitis with cerebral and subdural hematoma	Not clear
Allahyari et al. (2021) [26]	Case report	Iran	18-year-old, Female	Generalized tonic–colonic seizures	Bilateral pulmonary crackles, confusion, meningism, neck stiffness.	Positive IgM for COVID-19	CBC: ↑WBCs, neutrophil dominant, lymphopenia, ↑CRP. CSF: anti-NMDAR antibody. MRI: normal	Anti-NMDAR encephalitis with brain edema due to COVID-19	Discharged with full recovery after 2 months
Sattar et al. (2020) [27]	Case report	New York	44-year-old, Male	Fever (7 days), cough, SOB	Confusion, minimally responsive.	Positive PCR in NP swab	Chest X-ray: diffuse bilateral opacities. MRI: abnormal cortical signals in cortical frontal lobes.	Acute viral encephalitis secondary to SARS-CoV-2	Seizures stopped, discharged day 34
Braccia et al. (2021) [28]	Case report	Ferrara, Italy	70-year-old, Man	Fever, cough, SOB, confessional state	Right focal signs, vigilance fluctuations.	Positive PCR in NP swab	EEG: nonspecific mild background activity. Brain MRI: T2-FLAIR hyper intensity in the mesial temporal lobes.	Limbic encephalitis due to SARS-CoV-2	Improved cognition and alertness, MRI was similar after 2 months
Cheraghali et al. (2021) [29]	Case report	Tamin Ejtemae Hospital in Gonbad, Iran	34-month-old, Boy	Fever, tonic-clonic seizures, LOC	Upward gaze.	Positive PCR in NP swab	Brain MRI: symmetric, cortical, and juxta-cortical high T1 and T2 signal abnormality, in bilateral parieto-occipital lobes.	Viral SARS-CoV-2 encephalitis, with possible parenchymal hemorrhagic components	Decerebrate posture, ventilator independent then discharged
de Freitas et al. (2021) [30]	Case report	Rio de Janeiro, Brazil	35-year-old male, Man	Fever, diarrhea, vomiting, diplopia, urinary retention, sleepiness, LOC, +Babinski sign	Somnolent, oriented, convergence strabismus, mild ataxia in arms, brisk deep tendon reflexes.	Negative PCR in NP swab	Brain CT: normal. EEG: normal. Ultrasound: DVT. Brain MRI: lesions on white matter hemispheres, the body and splenium of corpus callosum and cerebellar peduncles.	SARS-CoV-2-associated meningitis–encephalitis	Discharged 21 days after admission with diplopia and urinary retention
Demirci et al. (2020) [31]	Case report	Turkey	48-year-old, Male	Headache, cough (10 days), fatigue, myalgia (7 days)	Normal.	Negative PCR in NP swab	MRI: hyperintense lesions in the posterior medial temporal lobe and hyperintense lesions in upper cervical spinal cord.	Viral encephalomyelitis due to SARS-CoV-2	Stable and under treatment
Javidarabshahi et al. (2021) [32]	Case report	Iran	44-year-old, Male	Febrile, dizzy, convulsion, respiratory symptoms	Not mentioned.	Positive PCR in NP swab	Head contrast-enhanced MRI: revealed a normal image.	Severe acute COVID-19 encephalitis by CSF analysis	Not mentioned
Glavin et al. (2021) [33]	Case report	UK	35-year-old, Male	Dysphasia, confusion, right arm incoordination	Right arm weakness, dysphasia, amnesia, vomiting, pyrexia, GCS 15/15, no meningism.	Negative PCR in NP swab	MRA brain: normal with congenitally hypoplastic left A1 segment of ACA. Other neuroimaging: normal. EEG: excess slow waves.	COVID-19 encephalitis based on CSF and EEG	Full recovery then discharged.
Kamal et al. (2020) [34]	Case report	Dubai, UAE	31-year-old, Male	Mild cough	Afebrile, normal vitals, O2sat 100%, confusion, agitation, fluctuations in loc.	Positive PCR in NP swab	Uncontracted brain CT: Multiple hypodensities in the external capsules. Contrast brain MRI: abnormal signal intensity in the temporal lobe.	COVID-19 encephalitis confirmed by MRI	Patient discharged and given vitamin C tablets and zinc supplements
Matos et al. (2021) [35]	Case series	Brazil	47-year-old, Female	Headache, AMS, sleep disturbance, confusion	MMSE: 30/30, multimedia over Coaxial Alliance 24/30.	Positive PCR in NP swab	CBC and imaging: normal.	COVID-19 encephalopathy	Not mentioned
Oosthuizen et al. (2021) [36]	Case report	South Africa	52-year-old, Male	Gait instability	Pyrexial alerted and oriented, multidirectional nystagmus, dysarthria, truncal appendicular ataxia.	Negative PCR in NP swab	CBC: ↑WBCs, neutrophil predominant, ↑ESR. EEG: normal. MRI brain: brainstem encephalitis. Uncontracted CT brain: central midbrain hypodensity.	SARS-CoV-2 brainstem encephalitis	Discharged, CSF examination remained normal at 6 months
Pandey et al. (2021) [37]	Case report	Delhi, India	11-year-old, Boy	Fever, headache, vomiting, altered sensorium (1 day)	Stable with GCS 9/15. Neck stiffness, +Kernig’s sign, no cranial nerve paresis, increased tone with brisk reflexes and extensor planters in lower limbs.	Positive PCR in NP swab	CBC: severe lymphopenia. Head CECT: scan was normal.	Acute meningoencephalitis confirmed by CSF analysis	Discharged. at day10 of illness
Tuma et al. (2020) [38]	Case series	São Paulo, Brazil	50-year-old, Woman	Fever	Not mentioned.	Positive PCR in NP swab	CBC: ↑WBCs with ↑eosinophils. Brain CT: normal. No MRI.	COVID-19 encephalopathy	Not mentioned

Abbreviations: COVID-19, coronavirus disease 2019; CSF, cerebrospinal fluid; LOC, loss of consciousness; SOB, shortness of breath; O2sat, oxygen saturation level; GCS, Glasgow Coma Scale; MMSE, Mini-Mental State Examination; PCR, polymerase chain reaction; NP, nasopharyngeal; IgM, immunoglobulin M; CBC, complete blood count; LDH, lactate dehydrogenase; WBCs, white blood cells; CRP, C-reactive protein; CT, computed tomography; MRI, magnetic resonance imaging; FLAIR, fluid-attenuated inversion recovery; MRA, magnetic resonance angiography; CECT, contrast enhanced computed tomography.

**Table 2:** Time from onset of symptoms to SARS-CoV-2 testing with reported CT thresholds and genes tested.

Study	Symptoms to Positive NP Swab Collection	CT Threshold for SARS-CoV-2 NP Swab	Symptoms to Positive CSF Collection	CT Threshold for SARS-CoV-2 Positive CSF and Tested Genes
Matos et al. (2021) [35]	Not mentioned	Not mentioned	day 16	Not mentioned
Braccia et al. (2021) [28]	Not mentioned	Not mentioned	Not mentioned	Not mentioned
Glavin et al. (2021) [33]	Negative swab	Negative swab	4 days	E gene (CT value: 35.8) S gene (CT value: 35.7)
Allahyari et al. (2021) [26]	3 weeks	Not mentioned	3 weeks	Not mentioned
Cheraghali et al. (2021) [29]	26 days	PCR 1: E gene PCR 2 N: gene ORF1ab gene (all CT value: 29)	26 days	PCR 1: E gene PCR 2 N: gene, ORF1ab gene (all CT value: 29)
De Freitas et al. (2021) [30]	Negative swab	Negative swab	3 days	Not mentioned
Demirci et al. (2020) [31]	Negative swab	Negative swab	10 days	Not mentioned
Shahali et al. (2021) [21]	4 days	Not mentioned	4 days	Not mentioned
Javidarabshahi et al. (2021) [32]	7 days	Not mentioned	7 days	Not mentioned
Virhammar et al. (2020) [19]	7 days	Not mentioned	19 days	N gene (CT value: 34.2)
Oosthuizen et al. (2021) [36]	Negative swab	Negative swab	6 days	E gene (CT value: 33) RdRP gene (CT value: 34) N gene (CT value: 35)
Yousefi et al. (2021) [18]	Negative swab	Negative swab	3 days	Not mentioned
Al-olama et al. (2020) [25]	7 days	Not mentioned	20 days	Not mentioned
Pandey et al. (2021) [37]	1 days	Not mentioned	1 days	Not mentioned
Fadakar et al. (2020) [22]	13 days	Not mentioned	13 days	Not mentioned
Steininger et al. (2021) [20]	1 days	Not mentioned	3 days	E gene (CT value: 19.5) RdRP gene (CT value: 21.6)
Tuma et al. (2020) [38]	1 days	Not mentioned	12 days	Not mentioned
Domingues et al. (2020) [12]	Negative swab	Negative swab	3 weeks	RdRP-2 gene (CT not mentioned)
Sattar et al. (2020) [27]	7 days	Not mentioned	32 days	Not mentioned
Moriguchi et al. (2020) [11]	Negative swab	Negative swab	9 days	N gene (CT value: 37) N-2 gene negative
Huang et al. (2020) [23]	Not mentioned	Not mentioned	Not mentioned	Not mentioned
Kamal et al. (2020) [34]	5 days	Not mentioned	5 days	N gene, E gene, RdRP and ORF1ab
Khodamoradi et al. (2020) [24]	Negative swab	Negative swab	3 days	Not mentioned

Abbreviations: NP, nasopharyngeal; CT, cycle threshold; SARS-CoV-2, severe acute respiratory syndrome coronavirus 2; CSF, cerebrospinal fluid; E, envelope; S, spike; PCR, polymerase chain reaction; N, nucleocapsid; ORF1ab, replicase; RdRP, RNA-dependent RNA polymerase.

**Table 3:** CSF changes in cases with confirmed COVID-19 CSF presence.

Study	Color	Protein (mg/dL)	Glucose (mg/dL)	WBCs (Cells/µL)	Bacteria	Viruses	COVID-19	Other
Yousefi et al. (2021) [18]	Colorless, clear	81	51	1870	Negative	Negative	PCR positive	
Virhammar et al. (2020) [19]	Not described	95 (at day 7), 26 (at day 12)	Not mentioned	5	Negative	Negative	PCR positive on day 12	IL6, NfL, and tau increased. Oligoclonal bands present
Steininger et al. (2021) [20]	Not described	136	12	57	Negative	Negative	PCR (in-house method) positive on day 2, positive till day 20	3 RBCs/μL
Shahali et al. (2021) [21]	Not described	128	68	96	Negative	Negative	PCR positive	
Fadakar et al. (2020) [22]	Not described	58	60	10 (80% lymphocytes)	Negative	Negative	PCR positive	
Domingues et al. (2020) [12]	Not described	32	68	1	Negative	Negative	PCR positive, confirmed by gene sequencing	
Huang et al. (2020) [23]	Not described	100	120	70 (100% lymphocytes)	Negative	Negative	PCR positive	65 RBCs
Moriguchi et al. (2020) [11]	Clear and colorless	Not mentioned	Not mentioned	9	Not mentioned	Negative	PCR positive	
Khodamoradi et al. (2020) [24]	Not described	0.2 (at day 1), 685 (at 1 week)	45	90	Negative	Negative	PCR positive	57 RBCs after 1 week
Sattar et al. (2020) [27]	Pink	39	75	Not mentioned	Negative	Negative	PCR positive	1685 RBCs
Allahyari et al. (2021) [26]	Light pink	241	55	27	Negative	Negative	PCR positive	RBCs: 1997, lymphocytes: 93%, PMN: 7%
Al-olama et al. (2020) [25]	Not described	Not mentioned	Not mentioned	Not mentioned	Not mentioned	Not mentioned	Fluid from the chronic subdural hematoma PCR positive	Not mentioned
Braccia et al. (2021) [28]	Not described	Not mentioned	Not mentioned	Not mentioned	Negative	Negative	PCR positive	ND
Cheraghali et al. (2021) [29]	Clear and colorless	Normal (levels are not described)	Normal	Not mentioned	Negative	Negative	PCR positive	ND
De Freitas et al. (2021) [30]	Not described	8.6 (at day 4), 6.6 (at day 6), 6.7 (at day 8)	57 (at day 4), 54 (at day 6), 63 (at day 8)	Not mentioned	Negative	Negative	PCR positive	Lymphocytic pleocytosis, oligoclonal bands, and increased IL6 levels
Demirci et al. (2020) [31]	Colorless and clear	0.04	90	Not mentioned	Negative	Negative	PCR positive	No cell detected microscopically
Javidarabshahi et al. (2021) [32]	Not described	0.0034	Not mentioned	Not mentioned	Negative	Negative	PCR positive	LDH 40 U/L
Glavin et al. (2021) [33]	Clear CSF	52	66:90 CSF to serum glucose ratio	134 (99% lymphocytes)	Negative	Negative	PCR positive	RBCs 20 × 106/L
Kamal et al. (2020) [34]	Clear and colorless	45	60	<5	Negative	Negative	PCR positive	CSF chloride: 119 mg/dL, RBCs: 50 cells/cm
Matos et al. (2021) [35]	Not described	Not mentioned	Not mentioned	Not mentioned	Negative	Negative	PCR positive	
Oosthuizen et al. (2021) [36]	Not described	37	64	51 (49 lymphocytes, 2 polymorphonuclear)	Negative	Negative	PCR positive	Increased IGg index, albumin (157 mg/L)
Pandey et al. (2021) [37]	Not described	696	Normal levels	75 pleocytosis (lymphocytic predominance 80%)	Negative	Negative	PCR positive	
Tuma et al. (2020) [38]	Not described	54	Not mentioned	15 (38% lymphocytes, 8% monocytes, 22% neutrophils, 31% eosinophils, and 1% macrophages)	Negative	Negative	PCR positive	IL6 of 19.57 pg/mL

Abbreviations: COVID-19, coronavirus disease 2019; CSF, cerebrospinal fluid; WBCs, white blood cells; RBCs, red blood cells; PCR, polymerase chain reaction; IgG, immunoglobulin G; LDH, lactate dehydrogenase; PMN, polymorphonuclear neutrophils; IL6, interleukin 6; NfL, neurofilament light.

## Data Availability

All data generated or analyzed during this study are included in this article and its Supplementary Material files. Further inquiries can be directed to the corresponding author.

## Supplementary Materials

The following supporting information can be downloaded at: https://www.mdpi.com/article/10.3390/cells11203212/s1, Table S1: Prisma checklist, search strategy, Table S2: Excluded articles at full-text screening, Table S3: Quality assessment. References [11,12,18,19,20,21,22,23,24,25,26,27,28,29,30,31,32,33,34,35,36,37,38,45,48,55,71,72,73,74,75,76,77,78,79,80,81,82,83,84,85,86,87,88,89,90] are cited in Supplementary Materials.
