# Supplementary material for: Clinical Implications of COVID-19 Presence in CSF: Systematic Review of Case Reports
Source: Cells. 2022 Oct 13;11(20):3212. doi: 10.3390/cells11203212 (PMC9600635; doi:10.3390/cells11203212)
Supplement: Supplementary file 1 [file cells-11-03212-s001.zip › cells-1941472-supplementary.pdf]

Supplementary material

Clinical Implications of Severe Acute Respiratory Syndrome Coronavirus 2 Presence in Cerebrospinal Fluid: Systematic Review of Case Reports

Ibrahim Elmakaty, Khaled Ferih, Omar Karen, Amr Ouda, Ahmed Alsa Elsabagh, Ahmed Amarah, Mohammed Imad Malki

Table of Contents

Table S1: Prisma checklist

Search strategy

Table S2: Excluded articles at full-text screening

Table S3: Quality assessment

References

Table S1: Prisma checklist

Section and Topic	Item #	Checklist item	Location where item is reported
**TITLE**			
Title	1	Identify the report as a systematic review.	1
**ABSTRACT**			
Abstract	2	See the PRISMA 2020 for Abstracts checklist.	2
**INTRODUCTION**			
Rationale	3	Describe the rationale for the review in the context of existing knowledge.	3
Objectives	4	Provide an explicit statement of the objective(s) or question(s) the review addresses.	4
**METHODS**			
Eligibility criteria	5	Specify the inclusion and exclusion criteria for the review and how studies were grouped for the syntheses.	5
Information sources	6	Specify all databases, registers, websites, organisations, reference lists and other sources searched or consulted to identify studies. Specify the date when each source was last searched or consulted.	4, Supplementary material
Search strategy	7	Present the full search strategies for all databases, registers and websites, including any filters and limits used.	Supplementary material
Selection process	8	Specify the methods used to decide whether a study met the inclusion criteria of the review, including how many reviewers screened each record and each report retrieved, whether they worked independently, and if applicable, details of automation tools used in the process.	5
Data collection process	9	Specify the methods used to collect data from reports, including how many reviewers collected data from each report, whether they worked independently, any processes for obtaining or confirming data from study investigators, and if applicable, details of automation tools used in the process.	5
Data items	10a	List and define all outcomes for which data were sought. Specify whether all results that were compatible with each outcome domain in each study were sought (e.g. for all measures, time points, analyses), and if not, the methods used to decide which results to collect.	5
	10b	List and define all other variables for which data were sought (e.g. participant and intervention characteristics, funding sources). Describe any assumptions made about any missing or unclear information.	5
Study risk of bias assessment	11	Specify the methods used to assess risk of bias in the included studies, including details of the tool(s) used, how many reviewers assessed each study and whether they worked independently, and if applicable, details of automation tools used in the process.	5
Effect measures	12	Specify for each outcome the effect measure(s) (e.g. risk ratio, mean difference) used in the synthesis or presentation of results.	6
Reporting bias assessment	14	Describe any methods used to assess risk of bias due to missing results in a synthesis (arising from reporting biases).	Not applicable

Certainty assessment	15	Describe any methods used to assess certainty (or confidence) in the body of evidence for an outcome.	Not applicable
**RESULTS**			
Study selection	16a	Describe the results of the search and selection process, from the number of records identified in the search to the number of studies included in the review, ideally using a flow diagram.	6, Figure 1
	16b	Cite studies that might appear to meet the inclusion criteria, but which were excluded, and explain why they were excluded.	Supplementary material
Synthesis methods	13a	Describe the processes used to decide which studies were eligible for each synthesis (e.g. tabulating the study intervention characteristics and comparing against the planned groups for each synthesis (item #5)).	Not applicable
	13b	Describe any methods required to prepare the data for presentation or synthesis, such as handling of missing summary statistics, or data conversions.	Not applicable
	13c	Describe any methods used to tabulate or visually display results of individual studies and syntheses.	6
	13d	Describe any methods used to synthesize results and provide a rationale for the choice(s). If meta-analysis was performed, describe the model(s), method(s) to identify the presence and extent of statistical heterogeneity, and software package(s) used.	6
	13e	Describe any methods used to explore possible causes of heterogeneity among study results (e.g. subgroup analysis, meta-regression).	Not applicable
	13f	Describe any sensitivity analyses conducted to assess robustness of the synthesized results.	Not applicable
Study characteristics	17	Cite each included study and present its characteristics.	Table-1
Risk of bias in studies	18	Present assessments of risk of bias for each included study.	Supplementary material
Results of individual studies	19	For all outcomes, present, for each study: (a) summary statistics for each group (where appropriate) and (b) an effect estimate and its precision (e.g. confidence/credible interval), ideally using structured tables or plots.	Not applicable
Results of syntheses	20a	For each synthesis, briefly summarise the characteristics and risk of bias among contributing studies.	7
	20b	Present results of all statistical syntheses conducted. If meta-analysis was done, present for each the summary estimate and its precision (e.g. confidence/credible interval) and measures of statistical heterogeneity. If comparing groups, describe the direction of the effect.	Not applicable
	20c	Present results of all investigations of possible causes of heterogeneity among study results.	Not applicable
	20d	Present results of all sensitivity analyses conducted to assess the robustness of the synthesized results.	Not applicable
Reporting biases	21	Present assessments of risk of bias due to missing results (arising from reporting biases) for each synthesis assessed.	Not applicable
Certainty of evidence	22	Present assessments of certainty (or confidence) in the body of evidence for each outcome assessed.	Not applicable

**DISCUSSION**			
Discussion	23a	Provide a general interpretation of the results in the context of other evidence.	10
	23b	Discuss any limitations of the evidence included in the review.	13
	23c	Discuss any limitations of the review processes used.	13
	23d	Discuss implications of the results for practice, policy, and future research.	12
**OTHER INFORMATION**			
Registration and protocol	24a	Provide registration information for the review, including register name and registration number, or state that the review was not registered.	4
	24b	Indicate where the review protocol can be accessed, or state that a protocol was not prepared.	4
	24c	Describe and explain any amendments to information provided at registration or in the protocol.	4
Support	25	Describe sources of financial or non-financial support for the review, and the role of the funders or sponsors in the review.	14
Competing interests	26	Declare any competing interests of review authors.	14
Availability of data, code and other materials	27	Report which of the following are publicly available and where they can be found: template data collection forms; data extracted from included studies; data used for all analyses; analytic code; any other materials used in the review.	14

Search strategy

Pubmed: Final search run on 19/12/2021

(COVID-19[Mesh] OR “COVID 19”[tiab] OR “Coronavirus Disease 2019”[tiab] OR “Coronavirus Disease-19”[tiab] OR “Coronavirus Disease 19”[tiab] OR “SARS-CoV-2 Infection*”[tiab] OR “2019-nCoV Infection*”[tiab] OR “2019 nCoV infection*”[tiab] OR “SARS Coronavirus 2 Infection*”[tiab] OR “2019 Novel Coronavirus Disease” [tiab] OR “2019 Novel Coronavirus Infection*”[tiab])

AND

(Central Nervous System Infections[Mesh] OR Meningitis[Mesh] OR Encephalitis[Mesh] OR Meningoencephalitis[Mesh] OR “Brain Infection*”[tiab] OR “Encephalomeningitis”[tiab] OR “Brain Fever”[tiab] OR “Meningoencephalitis”[tiab] OR “Cerebrospinal Fever”[tiab] OR “Choriomeningitis”[tiab] OR “Cerebromeningitis”[tiab] OR “Leptomeningitis”[tiab])

AND

(“Case Reports”[pt] OR “Case Report”[tiab] OR “Case series”[tiab])

NOT

(Animals[Mesh] NOT (Animals[Mesh] AND Humans[Mesh]))

NOT

(Randomized controlled trial[pt] OR Editorial[pt] OR Meta-Analysis[pt] OR “Systematic Review”[pt])

Embase: Final search run on 19/12/2021

(coronavirus disease 2019/exp OR "COVID 19":ti,ab OR "Coronavirus Disease 2019":ti,ab OR "Coronavirus Disease-19":ti,ab OR "Coronavirus Disease 19":ti,ab OR "SARS-CoV-2 Infection*":ti,ab OR "2019-nCoV Infection*":ti,ab OR "2019 nCoV infection*":ti,ab OR "SARS Coronavirus 2 Infection*":ti,ab OR "2019 Novel Coronavirus Disease":ti,ab OR "2019 Novel Coronavirus Infection*":ti,ab)
AND
(" central nervous system infection "/exp OR meningitis/exp OR encephalitis/exp OR meningoencephalitis/exp OR "Brain Infection*":ti,ab OR Encephalomeningitis:ti,ab OR "Brain Fever":ti,ab OR Meningoencephalitis:ti,ab OR "Cerebrospinal Fever":ti,ab OR Choriomeningitis:ti,ab OR Cerebromeningitis:ti,ab OR Leptomeningitis:ti,ab)
AND
(term:it OR "Case Report":ti,ab OR "Case series":ti,ab)
NOT
(animal/exp NOT (animal/exp AND human/exp))

Web of science: Final search run on 19/12/2021

(COVID-19 OR "COVID 19" OR "Coronavirus Disease 2019" OR "Coronavirus Disease-19" OR "Coronavirus Disease 19" OR "SARS-CoV-2 Infection*" OR "2019-nCoV Infection*" OR "2019 nCoV infection*" OR "SARS Coronavirus 2 Infection*" OR "2019 Novel Coronavirus Disease" OR "2019 Novel Coronavirus Infection*")

AND

("Central Nervous System Infections" OR Meningitis OR Encephalitis OR Meningoencephalitis OR "Brain Infection*" OR Encephalomeningitis OR "Brain Fever" OR Meningoencephalitis OR "Cerebrospinal Fever" OR Choriomeningitis OR Cerebromeningitis OR Leptomeningitis)

AND

("Case Reports" OR "Case Report" OR "Case series")

NOT

(Animals NOT (Animals AND Humans))

NOT

("Randomized controlled trial" OR Editorial OR Meta-Analysis OR "Systematic Review")

Scopus: Final search run on 19/12/2021

(INDEXTERMS(COVID-19) OR TITLE-ABS("COVID 19") OR TITLE-ABS("Coronavirus Disease 2019") OR TITLE-ABS("Coronavirus Disease-19") OR TITLE-ABS("Coronavirus Disease 19") OR TITLE-ABS("SARS-CoV-2 Infection*") OR TITLE-ABS("2019-nCoV Infection*") OR TITLE-ABS("2019 nCoV infection*") OR TITLE-ABS("SARS Coronavirus 2 Infection*") OR TITLE-ABS("2019 Novel Coronavirus Disease") OR TITLE-ABS("2019 Novel Coronavirus Infection*"))
AND
(INDEXTERMS("Central Nervous System Infections") OR INDEXTERMS(Meningitis) OR INDEXTERMS(Encephalitis) OR INDEXTERMS(Meningoencephalitis) OR TITLE-ABS("Brain Infection*") OR TITLE-ABS(Encephalomeningitis) OR TITLE-ABS("Brain Fever") OR TITLE-ABS(Meningoencephalitis) OR TITLE-ABS("Cerebrospinal Fever") OR TITLE-ABS(Choriomeningitis) OR TITLE-ABS(Cerebromeningitis) OR TITLE-ABS(Leptomeningitis))
AND
(DOCTYPE("Case Reports") OR TITLE-ABS("Case Report") OR TITLE-ABS("Case series"))
AND NOT
(INDEXTERMS(Animals) AND NOT (INDEXTERMS(Animals) AND INDEXTERMS(Humans)))
AND NOT
(DOCTYPE("Randomized controlled trial") OR DOCTYPE(Editorial) OR DOCTYPE(Meta-Analysis) OR DOCTYPE("Systematic Review"))

Medrxiv and Biorxiv: Final search run on 21/12/2021

(Nervous System Infections OR Meningitis OR Encephalitis) AND Case report AND COVID-19

Table S2: Excluded articles at full-text screening

Study	Title	Reason for exclusion
Rethaningsih el al. (2021)[1]	Meningoencephalitis due to SARS-CoV-2 and tuberculosis co-infection: a case report from Indonesia	co-infection with TB (TB meningitis)
Neumann et al. (2020)[2]	Cerebrospinal fluid findings in COVID-19 patients with neurological symptoms	Negative RT-PCR for SARS-CoV-2 in CSF
Novi et al. (2020)[3]	COVID-19 in a MS patient treated with ocrelizumab: does immunosuppression have a protective role?	No COVID-19 CSF testing
Garg et al. (2020)[4]	Encephalopathy in patients with COVID-19: A review	wrong study design
Miqdad etal. (2021)[5]	COVID-19-Induced Encephalitis: A Case Report of a Rare Presentation With a Prolonged Electroencephalogram	No COVID-19 CSF testing
Naz et al. (2020)[6]	Meningitis as an Initial Presentation of COVID-19: A Case Report	No COVID-19 CSF testing
Pilotto et al. (2020)[7]	Clinical Presentation and Outcomes of Severe Acute Respiratory Syndrome Coronavirus 2-Related Encephalitis: The ENCOVID Multicenter Study	No COVID-19 CSF testing
Razzack et al. (2020)[8]	Acute disseminated encephalomyelitis and COVID-19: A Systematic review of Case-Reports and Case-Series	wrong study design
Basher et al. (2021)[9]	Aseptic Meningitis after Recovery from SARS-CoV-2 in an Allogeneic Stem Cell Transplant Recipient	Epstein-Barr virus (EBV) DNA was detected in CSF
Affes et al. (2021)[10]	COVID-19 Presenting With Confusion: An Unusual but Suggestive Electroencephalography Pattern of Encephalitis	No COVID-19 CSF testing
Vraka et al. (2021)[11]	Two Paediatric Patients with Encephalopathy and Concurrent COVID-19 Infection: Two Sides of the Same Coin?	Negative RT-PCR for SARS-CoV-2 in CSF
Umanah et al. (2021)[12]	Acute psychosis in association of COVID19 infection: A case report	no CNS infection
McCuddy et al. (2020)[13]	Acute Demyelinating Encephalomyelitis (ADEM) in COVID-19 infection: A Case Series	CSF was negative for Covid-19
Zhang et al. (2020)[14]	COVID-19-Associated Acute Disseminated Encephalomyelitis – A Case Report	CSF was negative for Covid-19
Li et al. (2021)[15]	Diagnosis and analysis of unexplained cases of childhood encephalitis in Australia using metagenomic next-generation sequencing	CSF was negative for Covid-19
Bodro et al. (2020)[16]	Increased CSF levels of IL-1β, IL-6, and ACE in SARS-CoV-2-associated encephalitis	CSF was negative for Covid-19
de Oliveira et al. (2020)[17]	Headache and pleocytosis in CSF associated with COVID-19: case report	No COVID-19 CSF testing
Ghosh et al. (2020)[18]	SARS-CoV-2-Associated Acute Hemorrhagic, Necrotizing Encephalitis (AHNE) Presenting with Cognitive Impairment in a 44-Year-Old Woman without Comorbidities: A Case Report	No COVID-19 CSF testing
Gunawardhana et al. (2021)[19]	Delayed presentation of postinfectious encephalitis associated with SARS-CoV-2 infection: a case report	No COVID-19 CSF testing
Høy Marbjerg et al. (2021)[20]	Possible Involvement of Central Nervous System in COVID-19 and Sequence Variability of SARS-CoV-2 Revealed in Autopsy Tissue Samples: A Case Report	Co-infection with Staphylococcus capitis.
Huo et al. (2021)[21]	Clinical features of SARS-CoV-2-associated encephalitis and meningitis amid COVID-19 pandemic	wrong study design
Maury et al. (2021)[22]	Neurological manifestations associated with SARS-CoV-2 and other coronaviruses: A narrative review for clinicians	wrong study design

Aden et al. (2021)[23]	CSF Biomarkers in Patients With COVID-19 and Neurologic Symptoms A Case Series	Negative RT-PCR for SARS-CoV-2 in CSF

Table S3: Quality assessment

Study	Does the patient(s) represent(s) the whole experience of the investigator (centre) or is the selection method unclear to the extent that other patients with similar presentation may not have been reported?	Was the exposure (COVID-19) adequately ascertained?	Was the outcome (CSF analysis) adequately ascertained?	Was follow-up long enough for outcomes to occur?	Is the case(s) described with sufficient details to allow other investigators to replicate the research or to allow practitioners make inferences related to their own practice?	risk of bais
Yousefi et al. (2021)[24]	Yes	Yes	Yes	No	Yes	Low risk
Virhammar et al. (2020)[25]	Yes	Yes	No	Yes	Yes	Low risk
Steininger et al. (2021)[26]	Yes	Yes	Yes	Yes	Yes	Low risk
Shahali et al. (2021)[27]	Yes	Yes	Yes	Yes	Yes	Low risk
Fadakar et al. (2020)[28]	Yes	Yes	Yes	Yes	No	Low risk
Domingues et al. (2020)[29]	No	Yes	Yes	No	No	High risk
Huang et al. (2020)[30]	No	Yes	No	Yes	No	High risk
Moriguchi et al. (2020)[31]	Yes	Yes	No	No	No	High risk
Khodamoradi et al. (2020)[32]	Yes	Yes	No	Yes	Yes	Low risk
Sattar et al. (2020)[33]	Yes	Yes	Yes	Yes	Yes	Low risk
Allahyari et al. (2021)[34]	No	Yes	Yes	Yes	Yes	Low risk
Al-olama et al. (2020)[35]	No	Yes	No	Yes	Yes	Medium risk
Braccia et al. (2021)[36]	Yes	No	No	Yes	No	High risk
Cheraghali et al. (2021)[37]	Yes	No	Yes	No	No	High risk
de Freitas et al. (2021)[38]	Yes	Yes	Yes	No	No	Medium risk
Demirci et al. (2020)[39]	Yes	Yes	Yes	Yes	No	Low risk
Javidarabshahi et al. (2021)[40]	Yes	No	No	Yes	Yes	Medium risk

Glavin et al. (2021)[41]	No	Yes	Yes	Yes	No	Medium risk
Kamal et al. (2020)[42]	Yes	No	No	Yes	Yes	Medium risk
Matos et al. (2021)[43]	Yes	Yes	Yes	Yes	Yes	Low risk
Oosthuizen et al. (2021)[44]	Yes	No	No	Yes	Yes	Medium risk
Pandey et al. (2021)[45]	Yes	Yes	Yes	No	Yes	Low risk
Tuma et al. (2020)[46]	Yes	Yes	No	No	No	High risk

Reference list

1. Rethaningsih, Purwaamijaya B, Tugasworo D, et al. Meningoencephalitis due to SARS-CoV-2 and tuberculosis co-infection: a case report from Indonesia. *BALI MEDICAL JOURNAL.* 2021;10(2):673-676.
2. Neumann B, Schmidbauer ML, Dimitriadis K, et al. Cerebrospinal fluid findings in COVID-19 patients with neurological symptoms. *Journal of the neurological sciences.* 2020;418:117090-117090.
3. Novi G, Mikulska M, Briano F, et al. COVID-19 in a MS patient treated with ocrelizumab: does immunosuppression have a protective role? *Multiple sclerosis and related disorders.* 2020;42:102120-102120.
4. Garg RK, Paliwal VK, Gupta A. Encephalopathy in patients with COVID-19: A review. *Journal of medical virology.* 2021;93(1):206-222.
5. Miqdad MA, Enabi S, Alshurem M, Al-Musawi T, Alamri A. COVID-19-Induced Encephalitis: A Case Report of a Rare Presentation With a Prolonged Electroencephalogram. *CUREUS.* 2021;13(4).
6. Naz S, Hanif M, Haider MA, Ali MJ, Ahmed MU, Saleem S. Meningitis as an Initial Presentation of COVID-19: A Case Report. *Front Public Health.* 2020;8:474.
7. Pilotto A, Masciocchi S, Volonghi I, et al. Clinical Presentation and Outcomes of Severe Acute Respiratory Syndrome Coronavirus 2-Related Encephalitis: The ENCOVID Multicenter Study. *Journal of Infectious Diseases.* 2021;223(1):28-37.
8. Razzack AA, Kassandra-Coronel M, Domingo PI. Acute disseminated encephalomyelitis and COVID-19: A Systematic review of Case-Reports and Case-Series. *Neurology.* 2021;96(15).
9. Basher F, Camargo JF, Diaz-Paez M, Lekakis LJ, Pereira DL. Aseptic Meningitis after Recovery from SARS-CoV-2 in an Allogeneic Stem Cell Transplant Recipient. *CLINICAL MEDICINE INSIGHTS-CASE REPORTS.* 2021;14.
10. Affes Z, Bouvard EJ, Levy P, Dussaule C, Grateau G, Haymann JP. COVID-19 Presenting With Confusion: An Unusual but Suggestive Electroencephalography Pattern of Encephalitis. *J Clin Neurophysiol.* 2021;38(3):e11-e13.
11. Vraka K, Ram D, West S, et al. Two Paediatric Patients with Encephalopathy and Concurrent COVID-19 Infection: Two Sides of the Same Coin? *Case Rep Neurol Med.* 2021;2021:6658000.
12. Umanah T, Arshad H, Noor E. Acute psychosis in association of COVID19 infection: A case report. *Neurology.* 2021;96(15).
13. McCuddy M, Kelkar P, Zhao Y, Wicklund D. Acute Demyelinating Encephalomyelitis (ADEM) in COVID-19 infection: A Case Series. *medRxiv.* 2020:2020.2007.2015.20126730.

14. Zhang T, Rodricks MB, Hirsh E. COVID-19-Associated Acute Disseminated Encephalomyelitis – A Case Report. *medRxiv.* 2020:2020.2004.2016.20068148.
15. Li C-X, Burrell R, Dale RC, et al. Diagnosis and analysis of unexplained cases of childhood encephalitis in Australia using metagenomic next-generation sequencing. *bioRxiv.* 2021:2021.2005.2010.443367.
16. Bodro M, Compta Y, Llansó L, et al. Increased CSF levels of IL-1β, IL-6, and ACE in SARS-CoV-2-associated encephalitis. *Neurol Neuroimmunol Neuroinflamm.* 2020;7(5).
17. de Oliveira FAA, Palmeira DCC, Rocha-Filho PAS. Headache and pleocytosis in CSF associated with COVID-19: case report. *Neurol Sci.* 2020;41(11):3021-3022.
18. Ghosh R, Dubey S, Finsterer J, Chatterjee S, Ray BK. SARS-CoV-2-Associated Acute Hemorrhagic, Necrotizing Encephalitis (AHNE) Presenting with Cognitive Impairment in a 44-Year-Old Woman without Comorbidities: A Case Report. *Am J Case Rep.* 2020;21:e925641.
19. Gunawardhana C, Nanayakkara G, Gamage D, et al. Delayed presentation of postinfectious encephalitis associated with SARS-CoV-2 infection: a case report. *NEUROLOGICAL SCIENCES.* 2021;42(9):3527-3530.
20. Høy Marbjerg L, Jacobsen C, Fonager J, et al. Possible Involvement of Central Nervous System in COVID-19 and Sequence Variability of SARS-CoV-2 Revealed in Autopsy Tissue Samples: A Case Report. *Clinical Pathology.* 2021;14.
21. Huo L, Xu KL, Wang H. Clinical features of SARS-CoV-2-associated encephalitis and meningitis amid COVID-19 pandemic. *World Journal of Clinical Cases.* 2021;9(5):1058-1078.
22. Maury A, Lyoubi A, Peiffer-Smadja N, de Broucker T, Meppiel E. Neurological manifestations associated with SARS-CoV-2 and other coronaviruses: A narrative review for clinicians. *Rev Neurol (Paris).* 2021;177(1):51-64.
23. Eden A, Kanberg N, Gostner J, et al. CSF Biomarkers in Patients With COVID-19 and Neurologic Symptoms A Case Series. *NEUROLOGY.* 2021;96(2):E294-E300.
24. Yousefi K, Poorbarat S, Abasi Z, Rahimi S, Khakshour A. Viral Meningitis Associated With COVID-19 in a 9-year-old Child: A Case Report. *PEDIATRIC INFECTIOUS DISEASE JOURNAL.* 2021;40(2):E87-E88.
25. Virhammar J, Kumlien E, Fällmar D, et al. Acute necrotizing encephalopathy with SARS-CoV-2 RNA confirmed in cerebrospinal fluid. *Neurology.* 2020;95(10):445-449.
26. Steininger PA, Seifert F, Balk S, et al. Pearls & Oy-sters: SARS-CoV-2 Infection of the CNS in a Patient With Meningeosis Carcinomatosa. *Neurology.* 2021;96(10):496-499.
27. Shahali H, Ghasemi A, Farahani RH, Nezami Asl A, Hazrati E. Acute transverse myelitis after SARS-CoV-2 infection: a rare complicated case of rapid onset paraplegia. *J Neurovirol.* 2021;27(2):354-358.
28. Fadakar N, Ghaemmaghami S, Masoompour SM, et al. A First Case of Acute Cerebellitis Associated with Coronavirus Disease (COVID-19): a Case Report and Literature Review. *Cerebellum (London, England).* 2020;19(6):911-914.
29. Domingues RB, Mendes-Correa MC, de Moura Leite FBV, et al. First case of SARS-COV-2 sequencing in cerebrospinal fluid of a patient with suspected demyelinating disease. *Journal of neurology.* 2020;267(11):3154-3156.
30. Huang YH, Jiang D, Huang JT. SARS-CoV-2 Detected in Cerebrospinal Fluid by PCR in a Case of COVID-19 Encephalitis. *Brain, behavior, and immunity.* 2020;87:149-149.
31. Moriguchi T, Harii N, Goto J, et al. A first case of meningitis/encephalitis associated with SARS-Coronavirus-2. *International journal of infectious diseases : IJID : official publication of the International Society for Infectious Diseases.* 2020;94:55-58.

32. Khodamoradi Z, Hosseini SA, Gholampoor Saadi MH, Mehrabi Z, Sasani MR, Yaghoubi S. COVID-19 meningitis without pulmonary involvement with positive cerebrospinal fluid PCR. *European Journal of Neurology.* 2020;27(12):2668-2669.
33. Sattar SB, Haider MA, Zia ZS, Niazi M, Iqbal QZ. Clinical, Radiological, and Molecular Findings of Acute Encephalitis in a COVID-19 Patient: A Rare Case Report. *CUREUS.* 2020;12(9).
34. Allahyari F, Hosseinzadeh R, Nejad JH, Heiat M, Ranjbar R. A case report of simultaneous autoimmune and COVID-19 encephalitis. *JOURNAL OF NEUROVIROLOGY.* 2021;27(3):504-506.
35. Al-olama M, Rashid A, Garozzo D. COVID-19-associated meningoencephalitis complicated with intracranial hemorrhage: a case report. *ACTA NEUROCHIRURGICA.* 2020;162(7):1495-1499.
36. Braccia A, Carta F, Fiorillo D, et al. A case of limbic encephalitis with CSF detection of sars-cov2 virus: Immune-mediated mechanism or direct viral damage? *Journal of the Neurological Sciences.* 2021;429.
37. Cheraghali F, Tahamtan A, Hosseini SA, et al. Case Report: Detection of SARS-CoV-2 From Cerebrospinal Fluid in a 34-Month-Old Child With Encephalitis. *FRONTIERS IN PEDIATRICS.* 2021;9.
38. de Freitas GR, Figueiredo MR, Vianna A, et al. Clinical and radiological features of severe acute respiratory syndrome coronavirus 2 meningo-encephalitis. *Eur J Neurol.* 2021;28(10):3530-3532.
39. Demirci Otluoglu G, Yener U, Demir MK, Yilmaz B. Encephalomyelitis associated with Covid-19 infection: case report. *British Journal of Neurosurgery.* 2020:1-3.
40. Javidarabshahi Z, Najafi S, Raji S. Meningitis induced by severe acute respiratory syndrome coronavirus 2: A case report. *Iranian Red Crescent Medical Journal.* 2021;23(6).
41. Glavin D, Kelly D, Gallen B. COVID-19 encephalitis with SARS-CoV-2 detected in cerebrospinal fluid presenting as a stroke mimic. *European Stroke Journal.* 2021;6(1):481.
42. Kamal YM, Abdelmajid Y, Al Madani AAR. Cerebrospinal fluid confirmed COVID-19-associated encephalitis treated successfully. *BMJ Case Rep.* 2020;13(9).
43. Matos ADB, Dahy FE, De Moura JVL, et al. Subacute Cognitive Impairment in Individuals With Mild and Moderate COVID-19: A Case Series. *FRONTIERS IN NEUROLOGY.* 2021;12.
44. Oosthuizen K, Steyn EC, Tucker L, Ncube IV, Hardie D, Marais S. SARS-CoV-2 Encephalitis Presenting as a Clinical Cerebellar Syndrome: A Case Report. *Neurology.* 2021;97(1):27-29.
45. Pandey M. Acute Meningoencephalitis in a Child Secondary to SARS-CoV-2 Virus. *Indian Pediatr.* 2021;58(2):183-184.
46. Tuma R, Guedes B, Carra R, et al. Clinical, cerebrospinal fluid and neuroimaging findings in COVID-19 encephalopathy: a case series. *medRxiv.* 2020:2020.2008.2028.20181883.
